# In Search for a Planet Better than Earth: Top Contenders for a Superhabitable World

**DOI:** 10.1089/ast.2019.2161

**Published:** 2020-12-14

**Authors:** Dirk Schulze-Makuch, René Heller, Edward Guinan

**Affiliations:** ^1^ Astrobiology Group, Center for Astronomy and Astrophysics, Technische Universität Berlin, Berlin, Germany.; ^2^ GFZ German Research Center for Geosciences, Section Geomicrobiology, Potsdam, Germany.; ^3^ Leibniz-Institute of Freshwater Ecology and Inland Fisheries (IGB), Department of Experimental Limnology, Stechlin, Germany.; ^4^ School of the Environment, Washington State University, Pullman, Washington, USA.; ^5^ Max Planck Institute for Solar System Research, Göttingen, Germany.; ^6^ Institute for Astrophysics, Georg-August University, Göttingen, Germany.; ^7^ Department of Astrophysics and Planetary Science, Villanova University, Villanova, Pennsylvania, USA.

**Keywords:** Extrasolar terrestrial planets, Habitability, Planetary environments

## Abstract

The fact that Earth is teeming with life makes it appear odd to ask whether there could be other planets in our galaxy that may be even more suitable for life. Neglecting this possible class of “superhabitable” planets, however, could be considered anthropocentric and geocentric biases. Most important from the perspective of an observer searching for extrasolar life is that such a search might be executed most effectively with a focus on superhabitable planets instead of Earth-like planets. We argue that there could be regions of astrophysical parameter space of star-planet systems that could allow for planets to be even better for life than our Earth. We aim to identify those parameters and their optimal ranges, some of which are astrophysically motivated, whereas others are based on the varying habitability of the natural history of our planet. Some of these conditions are far from being observationally testable on planets outside the solar system. Still, we can distill a short list of 24 top contenders among the >4000 exoplanets known today that could be candidates for a superhabitable planet. In fact, we argue that, with regard to the search for extrasolar life, potentially superhabitable planets may deserve higher priority for follow-up observations than most Earth-like planets.

## 1. Introduction

Earth is our home planet and our only reference scale in regard to life in all aspects. We observe how intrinsically interwoven the biosphere on our planet is with the geosphere (Lovelock and Margulis, 1974), which is in stark contrast to our neighboring planets or moons. These worlds are either lifeless (*e.g.*, Mercury) or, in the event indigenous life exists, it does not appear to be a global phenomenon (*e.g.*, Mars; Klein, 1999). Given the diversity of biology and the large biomass on our planet, it may seem odd to ask whether there could be other planets in our galaxy or universe that are even more suitable for life than the one we live on (Heller and Armstrong, 2014; Heller, 2015). Yet, the reference to Earth as the most suitable planet for life could also be considered anthropocentric and geocentric and would run contrary to the *Copernican Principle*.

In fact, the natural history of Earth shows that habitability fluctuated quite significantly over geological eons. This does not only refer to major asteroid impacts or other calamities that wiped out large parts of the biosphere, but also long-lasting Snowball Earth Events, which resulted in subdued biospheres for millions of years (Ward and Brownlee, 2000). Large differences in the habitability of our planet were also present before and after the Great Oxygenation Event about 2.4 billion years ago, whereby the higher oxygenation content played a significant role in the development of aerobic metabolism and with it also the advent of complex life (Bains and Schulze-Makuch, 2016). As complex life, we understand multicellular macroscopic life that has similar function to animals, plants, and fungi on Earth.

With these caveats in mind, in comparing the potential habitability of extrasolar worlds to that of Earth, we use Earth's habitability as it is today—with all the biomass and biodiversity familiar to us.

## 2. Habitability Considerations

Habitability is usually understood as a planet's potential to develop and maintain environments hospitable to life (Cockell *et al.*, 2016). Thus, it is difficult and even impossible to measure habitability with our current knowledge. It is important to note that a planet might be habitable, but lifeless, because the origin of life never occurred. This might be so because the environmental constraints for the origin of life are much more stringent than for the persistence of life.

Thus, when we discuss the possibility of habitable or superhabitable planets, we do not necessarily assume that these planets host life and thus do not address (at least directly) the notoriously difficult question on how, where, and under what conditions life originates (Schulze-Makuch and Bains, 2017)—conditions that might be very different from those under which life thrives. Another noteworthy point is that the range of habitable niches on a planet could be much greater for alternative biochemistries of extraterrestrial life and could also exhibit a much larger variety of forms and functions than life on Earth (Schulze-Makuch, 2015). If molecules other than water (*e.g.*, ammonia) are considered as a solvent for an alien biochemistry, the types of possible habitats become highly speculative and increase enormously in quantity.

But this kind of assessment goes beyond the scope of this article, and we will restrict our analysis and discussion to life as we know it meaning life that is assembled from carbon-based building blocks and water as a solvent, operating roughly under the environmental conditions of life on Earth. These limits, based on our current knowledge, are summarized in Table 1.

**Table 1.** Approximate Environmental Limits for Life on Earth as Currently Known

Environmental parameter	Bacteria and archaeans	Eukaryotes including multicellular organisms	Example environments
Temperature	−18*°*C to 130*°*C, *Methanopyrus kandleri*, 122*°*C *for growth*; *Geogemma barossii*, 130*°*C for survival, bacterial growth at least down to about *−*18*°*C	*−*18*°*C to ∼105*°*C Pompeii worm (105*°*C), Himalayan midge and the yeast *Rhodoturula glutinis* at *−*18*°*C	Submarine hydrothermal systems, geothermal hot springs, brine pockets in sea ice at about *−*30*°*C, deep continental areas
pH	pH −0.5 to 13, acido-philic archaeans such as *Ferroplasma* sp. (∼pH 0); *Plectonema* (pH 13), *Natrobacterium* (pH 10.5)	pH 0 − 10, fungi such as *Cephalosporium* (pH 0), many species of protists and rotifers (pH 10)	Acid mine drainage, geothermal sulfurous sites (*e.g.*, Yellowstone) Soda lakes, peridotite-hosted hydrothermal systems (*e.g.*, Lost City vent)
Water activity	0.611–1.0, halophilic bacteria and archaea down to 0.611	0.605–1.0, xerophilic fungi such as *Xeromyces bisporus* down to 0.605	Deep-sea brines, soda lakes, evaporate ponds, dry soils and rocks, food with high solute content
Low O_2_ content	Any anaerobic bacteria or archaea such as methanogens (oxygen below detection limit)	Some fungi (chytrids), loricifera? High tolerance to low O_2_ also in some turtles and the Crucian carp (temporarily anaerobic)	Anoxic marine or lacustrine sediments, intestinal organs, early Earth environments
Pressure	0.7 kPa to 1680 MPa, many bacteria down to 0.7 kPa, *Shewanella oneidensis* and *Escherichia coli* strains at 1680 MPa up to 30 h, piezophilic and barophilic bacteria	0.7 kPa to 108 MPa, lichen *Pleopsidium chlorophanum* down to 0.7 kPa, high diversity of invertebrates and fishes in ocean trenches	Deep oceanic trenches such as the 11,100 m deep Marianas Trench, Martian surface conditions (based on laboratory experiments)
Radiation	At least 10,000—11,000 grays in *Deinococcus radiodurans*	At least 1000 grays in cockroach *Blatella germanica*	No natural source of radiation on Earth at levels tolerated *by D. radiodurans*
Chemical extremes	Cd 2–5 mM, bacteria and archaea; Ni 2.5 mM, Co 20 mM, Zn 12 mM, Cd 2.5 mM, *Ralstonia eutrophus*	Algae, for example, *Euglena* and *Chlorella* can grow in Cd, Zn, and Co at mM concentrations	Submarine hydrothermal vent fluids and sulfides; some high-metal containing lakes

Modified from Schulze-Makuch and Irwin (2018); with references from therein, and additional data from Sharma *et al.* (2002), Schuerger and Nicholson (2016), and de Vera *et al.* (2013).

Using Earth's history as a guide on how variations in planetary properties affect habitability and life will inadvertently give our analyses a strong earth-centered bias. However, we only have relevant information for our planet and life as we know it, and our challenge will be to distinguish observed patterns that are only valid for life on our planet from those patterns that are valid for life in general.

There is one additional challenge when evaluating superhabitability because, based on our understanding and definition, it refers to both biomass and biodiversity. Both of these parameters will be affected differently by planetary properties. To achieve the largest possible biomass, planetary properties have to be fine-tuned to a certain optimum, which we will attempt to estimate based on Earth's natural history. For a highest possible biodiversity, however, the variations in those properties are critical because these variations will drive natural selection to come up with better organismic adaptations and astounding biological innovations, which resulted, at least in the case of Earth, in the rise of complex ecosystems and a large biosphere. This includes animals that we consider intelligent. However, not every superhabitable planet may produce intelligent life because these variations might have either been too small for life to advance toward higher complexity or too large for life to keep up and instead become extinct.

Although it is difficult to provide an exact measure of the degree of habitability for individual planets (Barnes *et al.*, 2015; Bean *et al.*, 2015; Heller, 2020), we know at least some parameters that are critical to habitability. For example, a planet with an atmosphere is in general more habitable than one that has none, because any water on the surface would not be stable in liquid form and eventually be lost into space. A very obvious example can be found in our immediate interplanetary neighborhood; Earth and the Moon are planetary bodies within the solar habitable zone, but the one without an atmosphere is utterly lifeless.

Numerical 1D simulations of hydrodynamic atmospheric escape show that planets or moons in the HZ with surface gravities of at least about 1.5 m/s^2^ may hold on to a substantial atmosphere over billions of years (Arnscheidt *et al.*, 2019). For comparison, Earth's surface gravity is about 9.81 m/s^2^, whereas the Moon's surface gravity is roughly 1.62 m/s^2^, which puts it into a transitional regime. It could be that slightly different impact conditions from those that led to the supposed formation of the Moon (Canup and Asphaug, 2001) would have resulted in a more massive natural satellite around the Earth, which itself could have become marginally habitable.

All these reflections, however, do not exclude the presence of subsurface habitats that might exist on icy moons such as Europa, Enceladus, or Titan, but these are unlikely to be richer in biomass or biodiversity than a planet that also allows life on its planetary surface (Greenberg, 2010).

It is challenging to assess habitability in an astrobiological context, but several approaches have been proposed. These vary depending on the nature of life and focus of parameters evaluated. For example, Hoehler (2007) and Barnes *et al.* (2015) considered energy as primary criterion for the presence of life, whereas Heller *et al.* (2014) and Méndez (2015) proposed a biosphere resembling that of Earth. Schulze-Makuch and Irwin (2006) and Irwin *et al.* (2014) also included putative organisms with an alternative biochemistry and unusual solvent requirements. Bounama *et al.* (2007) and Irwin *et al.* (2014) considered complex life rather than only microbial life.

There have been several metrics proposed for assessing habitability, the oldest being the Habitat Suitability Index (HSI), which has been extensively used since 1982 to assess the biological value of a habitat (Stuber *et al.*, 1982; Soniat and Brody, 1988). A more recent one is the Planetary Habitability Index (PHI), which is based on parameters that are thought to be essential requirements for any form of life such as the presence of a stable substrate, available energy, appropriate chemistry, and a liquid solvent on the planetary body of interest (Schulze-Makuch *et al.*, 2011). The PHI for early Earth, at the time life originated on our planet, was set arbitrarily to 1.0, so any superhabitable planet would have a value of >1.0 in the PHI metrics.

A further refinement of the PHI is the Biological Complexity Index (BCI; Irwin *et al.*, 2014), which included the assessment of whether a planetary body likely contains complex macroscopic life. In addition to the parameters relevant to the PHI, the BCI also considers parameters that are thought to be critical for the evolution of a higher degree of biological complexity such as thermal and geophysical properties and age characteristics of a planetary body presumed to favor the evolution of complex life. However, although it is tempting to use metrices to prioritize planets for astrobiological investigations from thousands of candidates, there are also dangers with oversimplification as pointed out by Schulze-Makuch and Guinan (2016) and Tasker *et al.* (2017).

## 3. What Makes a Planet Superhabitable?

When we look for a planet more habitable than Earth, which we believe can be expressed in practical terms as a planet with higher biomass and higher biodiversity, we have to distinguish between stellar properties and planetary properties that are relevant to habitability. Complex life will have more stringent requirements on both properties than microbial life and complex life will likely be present for any planetary environment with a global biosphere, which is at least as diverse and prevalent as that of Earth. Thus, one feasible approach might be to search for biosignatures of complex life to identify superhabitable planets and moons.

### 3.1. Stellar properties

In regard to suitable stellar properties, we need to consider that different types of stars have different life spans and energy output, and dwarf stars of spectral type G similar to our Sun, referred to as dG stars, may not be the most suitable host stars for life on their planets because of their relatively short life spans (Kasting *et al.*, 1993). Since it took about 3.5 billion years on Earth until complex macroscopic life appeared, and about 4 billion years for technologically advanced life (us), life on many planets orbiting dG stars may simply run out of time. This will be even more likely for stars with a larger mass than our Sun (B, A, and F type dwarf stars), which have an even shorter life span.

Furthermore, studies of solar proxies of our Sun have shown that young dG stars rotate >10 times faster than dG stars near the age of our Sun, and have correspondingly high levels of magnetic dynamo-driven activity and very intense coronal X-ray and chromospheric FUV emissions (Guinan *et al.*, 2005), which makes the origin and early evolution of life challenging. Heller and Armstrong (2014) argued that the increased life span of stars with masses lower than one solar mass may allow inhabited planets to build up a higher biodiversity and possibly even a more complex ecosystem.

This argument would lift K- and M-dwarf stars into the realm of superhabitable planet host stars. A minimum limit for the mass of stars hosting superhabitable planets, however, is given by the tidal locking (Kasting *et al.*, 1993), loss of seasons (Heller *et al.*, 2011), early water loss (Luger and Barnes, 2015), and high-energy bursts experienced by planets in the habitable zone around M dwarf stars. Furthermore, since the HZ is located much closer around M stars, planets in that zone will experience much higher exposure rates to solar wind. All things combined, M dwarfs might not be the best places for superhabitable worlds to emerge.

Hence, K dwarf stars might well offer the most benign (long-lived and stable) environments for superhabitable planets. Using a simple model for the erosion of planetary atmospheres due to the stellar wind and for the biologically active UV irradiance, Lingam and Loeb (2018, 2019) came to a similar conclusion: So did Cuntz and Guinan (2016). Thus, from an objective perspective, stars similar to our Sun of the dwarf G (dG) type may not be the most suited to host superhabitable planets, but the lower mass dK stars may be, because many of the disadvantages of either dG and dM stars do not apply.

### 3.2. Planetary properties

With regard to planetary properties, many parameters affect habitability, and many of these are intrinsically interlinked. Moreover, at this time few, if any, of these habitability parameters are reliably known for any exoplanet. One of these is a planet's geophysical differentiation into core, mantle, and crust. This differentiation in conjunction with an efficient recycle mechanism such as plate tectonics provides the opportunity for multiple habitats and interfaces between those habitats. If the planet is more massive than Earth, it will have a larger surface area, and more living space near its surface will be available. Thus, such a planet is potentially able to support more biomass and a higher biodiversity (Heller and Armstrong, 2014).

A larger planetary mass will also indicate a larger amount of interior heating through radioactive decay, which means the planet could stay habitable for a longer amount of time. A larger mass planet with higher gravity would also retain a thicker atmosphere, which would make flight the preferred way of locomotion. On Earth, flight is used by many species as a preferred manner of locomotion, and on a planet with an even thicker atmosphere, that would be even more befitting. This would have advantages for the distribution of species and settlements of islands and continents. However, this relationship would only hold to a certain extent, because if planetary mass becomes too large, the planet might evolve into a gas giant or mini-Neptune retaining the light gases such as hydrogen or being an undifferentiated iron-rich body. Also, subsurface living space may be smaller because of larger gravity due to smaller pore volumes and because a sterilizing temperature would be reached at a lower planetary depth.

It is naturally difficult to determine the optimal mass of a superhabitable planet, but Heller and Armstrong (2014) speculated that planets with up to twice Earth's mass could have the potential of superhabitability. However, more recent research indicates that many of the exoplanets with two Earth masses are mini-Neptunes rather than rocky planets (Zeng *et al.*, 2016), so a more conservative estimate of up to about 1.5–1.6 Earth masses seems to be in order. Also, more massive planets could create a stagnant lid at their core-mantle boundaries, which would result in a reduced heat flow from the core and, therefore, could impede plate tectonics (Stamenković *et al.*, 2012; Noack and Breuer, 2014). This might prevent the planet from driving its carbon silicate cycle, which acts as a natural thermostat on Earth. Furthermore, more massive planets may readily convert into a Venus-type planet with more volcanic activity and outgassed volatiles. Dorn *et al.* (2018) and Noack *et al.* (2017) suggested that there is a mass limit until which planets can effectively outgas dense atmospheres and stay habitable.

Temperature is a critical variable for all aspects of biology. Global temperatures are determined mostly by the planetary body's location in space relative to the star it orbits, its endogenic activity, and its temperature variations mostly by the tilt of the planet's axis relative to its host star. Life requires a certain range of temperatures, which is dependent on its biochemistry, and complex life on Earth has a narrower range than microbial life (Table 1). No empirical evidence is available, however, on what that optimum is, aside from the case of life as we know it on Earth. Based on our experience from Earth, the highest biomass and biodiversity is present in tropical rainforests, and the least in cold polar regions (Brown, 1990, 2013; Kraft *et al.*, 2011).

Thus, higher temperatures than currently existing on Earth seem to be more favorable. The caveat is that the necessary moisture has to be available as well because inland deserts with low biomass and biodiversity are also common on our planet. One example is the early Carboniferous period, which was warmer and wetter (Raymond, 1985; Bardossy, 1994) on our planet than today, with so much biomass produced that we still harvest the organic deposits in the form of coal, oil, and natural gas from it. Thus, a slightly higher temperature, perhaps by 5°C—similar to that of the early Carboniferous time period—would provide more habitable conditions until some optimum is reached. However, this will depend on the biochemistry and physiology of the inhabiting organisms and the amount of water present.

As pointed out earlier, a higher water content in the form of more moisture and more clouds would be beneficial in principle, with the rainforests on Earth being again a good example. A higher water content (absolute humidity) in the atmosphere would also provide more protection from UV irradiation. However, Earth is already covered by about 71% with water, so it is hard to see how slightly more coverage would be a significant benefit. The more critical parameter is the distribution of land and ocean areas, particularly shallow-water areas where biological diversity and biomass are highest. The position of the land areas and their split up will also affect patterns of oceanic and atmospheric circulation (Borchert, 1953).

Thus, more evenly distributed land areas would provide more land–ocean interfaces compared with the other extreme, the Permian time period, which is known for Pangaea, the presence of a huge supercontinent and large inland deserts (Glennie, 1987; Abrantes *et al.*, 2016). This is a scenario of an extremely unequal land–water distribution in the natural history of our planet, which was the worst case for habitability on Earth. In this aspect, plate tectonics is crucial. Plate tectonics (together with erosion) is largely responsible for the topography of our planet, and certain rates could lead to more biologically active coastal regions. Plate tectonics is critical for nutrient recycling, and without such an efficient recycling mechanism, nutrients for a global biosphere would be rather quickly depleted. Thus, plate tectonics is, at least for Earth, essential for its high degree of habitability. The rates of plate tectonics (movement of planets and creation of new ocean floor) varied throughout Earth′s history, and we do not know what the optimal rate of plate tectonic activity is to maximize biomass and biodiversity.

Another important parameter that would generally favorably affect the habitability of a planet is time, or better termed the age of the planet in question. On Earth, it took nearly 4 billion years until complex organisms were common on our planet. Most of the time was expended when Earth's rocks were oxidized and the buildup of a high oxygen content in the atmosphere occurred, without which fast-moving macro-organisms—animals—would be unthinkable. If the time period to achieve this level of complexity and biodiversity is only roughly average, it would mean statistically that on many habitable planets this evolutionary jump would not be reached in the same time period, perhaps may not even be reached during the life time of the star (especially if the star is a dG star similar to ours).

Thus, we have an optimal age also in this category, and we do not know where it is, except to say that a slightly older planet has higher chances to be more habitable than Earth. If a planet is too old, exhaustion of internally generated heat may result in eventual cooling, with consequences for global temperatures and atmospheric composition (although tidal interactions might counteract the cooling of the interior). This is especially relevant for planets with lower mass than Earth such as is observed for Mars in our own Solar System. Furthermore, the older a planet is the higher chances that it experienced a calamity that would have drastically affected the biosphere such as a sterilizing or near-sterilizing impact or a nearby supernova explosion. We note here that the calamity would have to result in extreme devastation, because even large events detrimental to the biosphere might actually spur evolution and thus result in a faster pathway toward biological complexity. Based on our current understanding, we estimate that a planet with an age of 5–8 billion years should—on statistical grounds—generally be more habitable than Earth.

Earth is rather unique in our solar system in that it has such a large moon (with the exception of the Pluto-Charon system). The Moon provides stability to Earth's rotation axis and thus climate, and the major impact that created the Moon, might have delivered critical elements such as carbon, nitrogen, and sulfur to the early Earth (Grewal *et al.*, 2019). Recent research indicates that a moon may not be needed for a stable obliquity (Lissauer *et al.*, 2011) and that extreme obliquity variations may not be detrimental to life (Armstrong *et al.*, 2014). However, if Earth would have had the huge and chaotic variations in obliquity Mars had (Ward, 1973; Touma and Wisdom, 1993; Mellon and Jakosky, 1995), the result would be detrimental for life and possibly result in major mass extinctions.

Variations in environmental parameters are generally beneficial for evolutionary progress, but only to a degree before the resulting drastic changes would likely be too extreme and result in the extinction of life. An additional advantage of Earth having its large Moon is that it causes tidal flats on our planet (with addition of tidal influences of the Sun), which may have played a role in the origin of life (Westall *et al.*, 2013). The Moon is still moving away from Earth, and it was much closer to Earth billions of years ago, at the dawn of life. The continuous cycling of magma within Earth, and water masses on Earth's surface, would have a tremendous benefit for the development of life.

In fact, the PHI for early Earth was assigned a 1.0 compared with the 0.96 of Earth today because of the reduction of tidal forces (Schulze-Makuch *et al.*, 2011). Thus, we think that a planet with a moon larger than Earth's Moon or closer than the distance of Earth to today's Moon would, in general, be more habitable than Earth. With a very massive moon too close, however, Earth's habitability would likely have been worse than it has been due to the tidal locking imposed by the gravitational pull imposed by such a hypothetical moon. Once in tidal locking, the Earth's day length could be several times its contemporary value, which would have dramatic effects on the surface distribution of sunlight as an external source of energy.

One critical property of whether a planet is more habitable than Earth is its planetary history, which cannot be evaluated, especially for any exoplanet in the near future (Schulze-Makuch and Guinan, 2016). Were conditions conducive to the origin of life during the planet's or moon's early history? Did a cataclysmic event occur in its subsequent history, which wiped out all life and made a later origin of life impossible? In case of Earth, such a sterilizing event occurred with the impact that created our Moon; however, conditions were afterwards still amenable for the origin of life. But what if the event would have occurred 3 billion years later when Earth's atmosphere was already rich in oxygen?

Although we do not know what the exact conditions are needed for the origin of life, one prerequisite seems to be the availability of anaerobic environments in which the synthesis of organic molecules can occur. In either case, we will not be able to answer this question when observing an exoplanet or exomoon today billions of years after its formation. Thus, even if we find a planet that would appear to be more habitable than Earth based on the identified parameters here, it may not be so. In fact, our “ideal” planet may even be uninhabitable and devoid of life. Nevertheless, based on our previous discussion we can put together the key points of our MVP (Most Valuable Planet) when searching for a potentially superhabitable planet (Table 2).

**Table 2.** Most Valuable Planets—Planets That Might Be More Habitable Than Earth

• In orbit around a K dwarf star
• About 5–8 billion years old
• Up to1.5 more massive than Earth and about 10% larger than Earth
• Mean surface temperature about 5°C higher than on Earth
• Moist atmosphere with 25–30% O_2_ levels, the rest mostly inert gases (*e.g.*, N_2_)
• Scattered land/water distributed with lots of shallow water areas and archipelagos
• Large moon (1–10% of the planetary mass) at moderate distance (10–100 planetary radii)
• Has plate tectonics or similar geological/geochemical recycling mechanism as well as a strong protective geomagnetic field

Note: Some relevant parameters. For an assessment of which planetary properties will likely be directly observable with current and proposed space missions the reader is referred to Table 1 of Schulze-Makuch and Guinan (2016).

## 4. Finding a Superhabitable Planet

Do we have a candidate that would fit to be an MVP? For now, this question remains open because we cannot evaluate all items on our list. Current technology simply does not allow us, for example, to measure global temperatures on extrasolar planets anywhere close to the accuracy needed. Data are also currently lacking to calculate the PHI for any exoplanet. Nevertheless, we can say whether a planet is in the habitable zone with temperatures where liquid water could be present on the planetary surface. Also, we cannot determine the amount of land areas on a planet or whether it has plate tectonics, nor whether it is orbited by a large moon. However, future technologies may allow us to do so. The planned Starshade mission is a first significant step in this direction (Turnbull *et al.*, 2012).

**FIG. 1.**
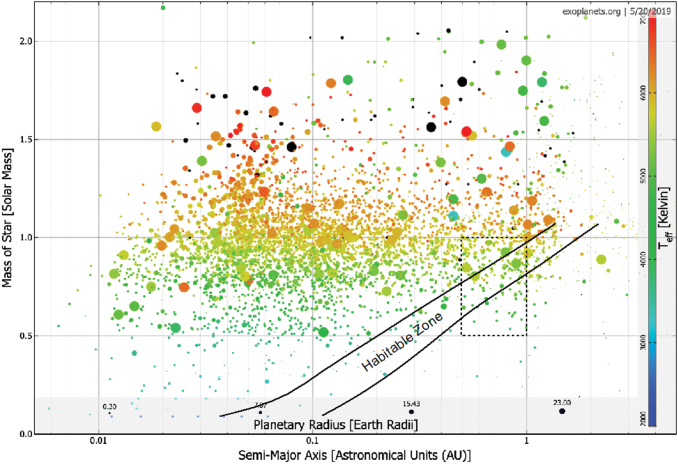
Star-planet distances (along the abscissa) and mass of the host star (along the ordinate) of roughly 4500 extrasolar planet and extrasolar planet candidates. The temperatures of the stars are indicated with symbol colors (see color bar). Planetary radii are encoded in the symbol sizes (see size scale at the bottom). The conservative habitable zone, defined by the moist-greenhouse and the maximum greenhouse limits (Kopparapu *et al.*, 2013) is outlined with black solid lines. Stellar luminosities required for the parameterization of these limits were taken from Baraffe *et al.* (2015) as a function of mass as shown along the ordinate of the diagram. The dashed box refers to the region shown in Fig. 2. Data from exoplanets.org as of May 20, 2019. Color images are available online.

**FIG. 2.**
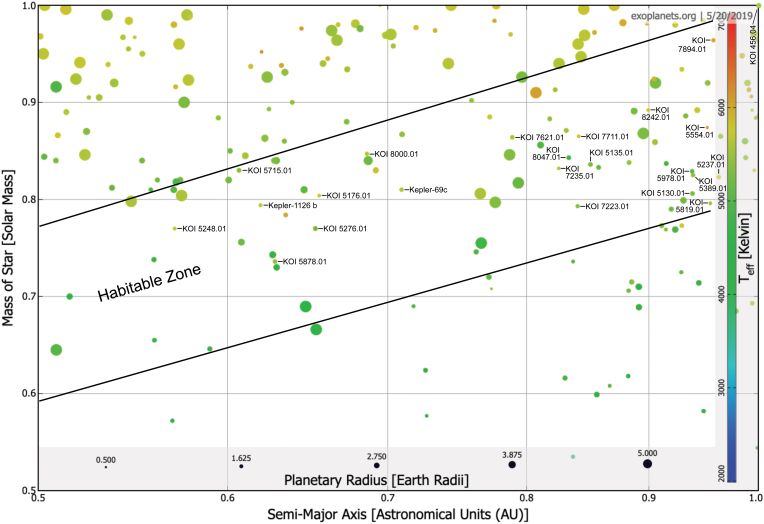
Same as Fig. 1, but here with a zoom into the habitable zone around K dwarf stars, the potential site for superhabitable planets. Twenty-four planets and planet candidates that are smaller than 2 Earth radii are labeled with name tags. Uncertainties in the observed stellar, planetary, and orbital parameters propagate into the planetary radius measurements, which is why we include planets as large as 2 Earth radii, although truly superhabitable planets might be restricted to radii <1.1 Earth radii. Color images are available online.

More than 4000 exoplanets and exoplanet candidates have been identified so far, and many of those are in the habitable zone (Fig. 1). This is a prerequisite of our selection process, or otherwise surface temperatures are unlikely to allow for a global biosphere. As discussed before, dK stars seem to have the ideal stellar properties to host superhabitable planets. Luckily, they are also relatively frequent in the galaxy (∼12%), more so than dG stars (∼ 8%). In the next step, the question naturally arises whether we can identify planets in the habitability zone of K dwarf stars. In a diagram spanning the star-planet distance and the stellar mass, the habitable zone is located between 0.5 and 1.0 AU and between 0.5 and 1.0 solar masses (Fig. 2).

Although an exact count of these potentially superhabitable planets is impossible given the uncertainties in our mostly qualitative model and given the uncertainties in the observed parameters, Fig. 2 shows that there are indeed at least about two dozen possible candidates for a superhabitable planet. We caution that we do not have any observational signatures of life from any of these planets. In fact, only Kepler 1126 b (KOI 2162) and Kepler-69c (KOI 172.02) are statistically validated planets (Morton *et al.*, 2016). The other objects are unconfirmed Kepler Objects of Interest (KOIs), some of which may turn out to be astrophysical false positives. Even Kepler-69c, whose planetary status has been statistically established, will likely not be the target of future follow-up observations with the James Webb Space Telescope or its successor, potentially LUVIOR. At a distance of almost 2000 light years, it is simply too far away. Our point here is not to identify potential targets for follow-up observations but to illustrate that superhabitable worlds may already be among the planets that have been detected.

A closer look at the 24 candidates in Fig. 2 reveals that 9 of them are orbiting around K stars, 16 of them are between about 5 and 8 billion years old (age estimates for the KOI samples are provided in Table 3 along with explanatory notes), and five of them are in the 10° range of the optimal temperature of a superhabitable planet as proposed by us (19°C; Table 3), with KOI-456.04 being the one with the most Earth-like temperature (Heller *et al.*, 2020b). Only one of the candidates (KOI 5715.01) fits all three criteria, but it has a predicted lower global temperature than Earth when a gray atmosphere model is used that includes an approximation for a greenhouse effect (Table 3). However, if the greenhouse effect is stronger than on Earth, KOI 5715.01 could conceivably be superhabitable.

**Table 3.** Stellar and Planetary Properties of Superhabitable Candidate Worlds as Identified in Fig. 2

													

Gray-shaded cells indicate superhabitable conditions as defined in the main text. Cells highlighted in light gray with print in bold with planetary radii <1.6$R_{⊕}$ indicate rocky composition of the planet (Rogers, 2015; Fulton *et al.*, 2017). KOI is the KOI Designation, KOI-2162.01 was statistically validated as planet Kepler-1126b and KOI-172.02 as Kepler-69c by Morton *et al.* (2016). G-mag is the G-magnitude from Gaia DR2 (Gaia Collaboration, 2016, 2018). Bp-Rp *blue-minus red* color index is also from the Gaia DR2 database (Gaia Collaboration, 2016, 2018). dist (kpc) is the distance in kiloparsecs from Gaia DR2 (Gaia Collaboration, 2016, 2018); calculated from the Gaia parallaxes (for KOI 5978 no Gaia parallax is available. *T*_eff_ (K) is the effective surface temperature of the star derived from the Gaia DR2 (Bp-Rp) index corrected for interstellar reddening as given in the Gaia DR2 database. Sp.Tp. (spectral type) is the spectral class of the star. The spectral type is estimated using Teff-spectral type given by Mamachek (2018). $L∕L_{⊙}$ is the stellar luminosity as a fraction of the solar luminosity as given in Gaia DR2 (Gaia Collaboration, 2016, 2018). $M∕M_{⊙}$ is the estimated mass of the star as a fraction of the solar mass—from Mamachek (2018) Mass-T_eff_- Spectral-type calibrations for main-sequence stars. Age (Gyr) is the approximate age of the star in Giga years (billion years), assumed to be also valid for the orbiting exoplanet, including error estimate. The age estimates are approximate based on stellar evolution codes plus indirect age estimates as additional note hereunder^*^. *P*_orb_ is the orbital period of the exoplanet around the star in days. $R∕R_{⊕}$ is the planetary radius as fraction of Earth radius based on minimum and maximum values given (including error) in the NASA Exoplanetary Archive (https://exoplanetarchive.ipac.caltech.edu). *a* (AU) is the distance of the star to the planet in astronomical units*. T*_p_ is the surface temperature of the planet (in °C) estimated from a gray atmosphere model (Heller et al., 2020a). This model encapsulates all atmospheric absorptive properties (including a greenhouse effect) in two coefficients, the planetary emissivity and the atmospheric optical depth (*τ*). The term “gray” refers to the neglect of any wavelength dependence in this model. *T*_p_ is calculated using *τ* = 0.35 as observed on Earth. For Earth, this parameterization yields *T*_p_ = 0.6°C for Earth, which is off by −13.4°C with respect to the actual global mean surface temperature. *T*_p_′ is the surface temperature of the planet (in °C) as for *T*_p_ but using *τ* = 0.705 to reproduce the Earth's global mean surface temperature of 14°C (Heller et al., 2020a). Additional Note on Stellar Ages^*^: Determining stellar ages is a notoriously difficult problem, even when good data are available. Exhaustive discussions of the various methods available have been given by Soderblom (2010). Preliminary age estimates for the KOI sample stars are provided in Table 3 along with the estimated age uncertainties. The initial stellar ages have been obtained using precise distances returned from Gaia DR2 (Gaia Collaboration, 2016, 2018; Andrae et al., 2018) as well as from Gaia photometry [G-magnitudes and (Bp-Rp)-colors]. The Gaia DR2 distances, G-magnitudes and (Bp-Rp) colors (Gaia Collaboration, 2016, 2018) are also provided in the table. The Gaia DR2 data of these stars are also provided in the *VIZIER* online database. The preliminary age estimates were constrained and improved by several indirect methods (Soderblom, 2010): The Gaia stellar magnitudes and photometric color indices have been corrected for Interstellar Medium (ISM) reddening and absorption in the published Gaia DR2 database (Gaia Collaboration, 2016, 2018) that also include temperatures and luminosities ($L∕L_{⊙}$) estimates. These are also given in Table 3. Absolute magnitudes, bolometric corrections, and reddening-corrected colors and $L∕L_{⊙}$ values are compared with the corresponding theoretical *M*_G_ (absolute Gaia magnitude) or luminosity—color (*T*_eff_) from stellar evolutionary models and isochrones. The *PARSEC* (Bressan *et al.*, 2012) and *MIST* (Dotter, 2016) models were utilized. The evolution models/isochrones also have been recently modified to handle Gaia DR2 photometry (*e.g.*, Howes and Bensby, 2017). Unfortunately, the reliability of evolutionary/isochronal ages is greatly hindered for the KOI sample by lack of accurate stellar temperatures and metal abundances. Solar abundances were assumed as a default when the stellar abundances were not available. However, from indirect age methods discussed by Soderblom (2010) and Guinan and Engle (2008), preliminary age estimates have been constrained and improved. Some of the age estimates from indirect methods include *Age–Rotation–Activity* relations, space velocities (tangential velocities), and photometric activity (old stars rotate slowly, are inactive, and tend to have very small brightness variations of <0.4%. Measures of stellar photometric activity and stellar rotation periods (if measured) are from McQuillan *et al.* (2014) and Mazeh *et al.* (2015). There are biases in this KOI data set that favor older, less photometrically active stars. This selection effect arises because these hosted earth-size planet candidates have very shallow transit eclipses and are less likely to be detected in younger photometrically “noisy” host stars. Another useful age indicator is the distance from the galactic plane (*Z*). The center of the Kepler field is at a galactic latitude *b* = +13.3°, because the majority of our KOI stars are faint with distances *d* > 900 pc, and are >200 pc above the galactic plane. These stars tend to be older stars and members of intermediate stellar disk population. In the future, more precise parallaxes from Gaia as well as improved chemical abundances and precise temperatures from spectroscopy should lead to better constrained ages.

One obvious parameter missing from Table 3 is the planetary mass. This lack of knowledge is inherent to most of the planets and candidates detected with the Kepler mission, which used the transit method. The transit method gives information about the radius of the planet relative to the stellar radius, but in most cases mass measurements required additional stellar spectroscopy follow-up observations to measure the radial velocity signature. Nevertheless, we highlight in Table 3 two planets with upper radius limit <1.6 $R_{⊕}$ that have a good potential of being rocky rather than gaseous planets (Rogers, 2015; Fulton *et al.*, 2017). As an interesting side note, we point out that superhabitable worlds are expected to be larger and more massive than Earth-sized, Earth-mass planets, and by virtue of the observational biases of the RV and transits methods, superhabitable planets should be easier to detect than Earth-like planets.

Some of the astrophysical conditions that we identify as crucial for a planet (or moon) to be potentially superhabitable are far from being observationally testable on planets outside the solar system. That said, our constraints allow us to distill a short list of top contenders among the >4000 exoplanets known today that could be candidates for a superhabitable planet or, more correctly, a superhabitable planet that follows the Earth model, because our analyses of the planetary properties is strongly based on Earth′s natural history. Our evaluation of stellar properties, however, does not have the strong Earth bias and has the result that planets orbiting K dwarf stars are more likely to be superhabitable and host life primarily due to the longer lifetimes of K-dwarfs relative to solar-type stars.

Although none of these planets and planet candidates is closer than 100 light years (Table 3) and, therefore, inaccessible for high-quality observations from NASA's TESS mission, we argue that superhabitable planets might well be present in the exoplanet sample known today. Should such a planet be discovered within about 100 light years in the near future, then such a world would deserve higher priority for follow-up observations in search of extrasolar life than the most Earth-like planets.

## Notes

Associate Editor: Christopher McKay

## Abbreviations Used

BCI: Biological Complexity Index
dG: dwarf G
KOI: Kepler Objects of Interest
MVP: most valuable planet
PHI: Planetary Habitability Index
